# Impairment of cell adhesion and migration by inhibition of protein disulphide isomerases in three breast cancer cell lines

**DOI:** 10.1042/BSR20193271

**Published:** 2020-10-23

**Authors:** Henry S. Young, Lucy M. McGowan, Katy A. Jepson, Josephine C. Adams

**Affiliations:** School of Biochemistry, University of Bristol, Bristol BS8 1TD, U.K.

**Keywords:** breast cancer, Cell attachment, Cell migration, Erp57, PDIA3, Secretome

## Abstract

Protein disulphide isomerase A3 (PDIA3) is an endoplasmic reticulum (ER)-resident disulphide isomerase and oxidoreductase with known substrates that include some extracellular matrix (ECM) proteins. PDIA3 is up-regulated in invasive breast cancers and correlates in a mouse orthotopic xenograft model with breast cancer metastasis to bone. However, the underlying cellular mechanisms remain unclear. Here we investigated the function of protein disulphide isomerases in attachment, spreading and migration of three human breast cancer lines representative of luminal (MCF-7) or basal (MDA-MB-231 and HCC1937) tumour phenotypes. Pharmacological inhibition by 16F16 decreased initial cell spreading more effectively than inhibition by PACMA-31. Cells displayed diminished cortical F-actin projections, stress fibres and focal adhesions. Cell migration was reduced in a quantified ‘scratch wound’ assay. To examine whether these effects might result from alterations to secreted proteins in the absence of functional PDIA3, adhesion and migration were quantified in the above cells exposed to media conditioned by wildtype (WT) or *Pdia3^−/−^* mouse embryonic fibroblasts (MEFs). The conditioned medium (CM) of *Pdia3^−/−^* MEFs was less effective in promoting cell spreading and F-actin organisation or supporting ‘scratch wound’ closure. Similarly, ECM prepared from HCC1937 cells after 16F16 inhibition was less effective than control ECM to support spreading of untreated HCC1937 cells. Overall, these results advance the concept that protein disulphide isomerases including PDIA3 drive the production of secreted proteins that promote a microenvironment favourable to breast cancer cell adhesion and motility, characteristics that are integral to tumour invasion and metastasis. Inhibition of PDIA3 or related isomerases may have potential for anti-metastatic therapies.

## Introduction

Breast cancer accounts for 15% of new cancer cases each year in the U.K. [1]. It has the second highest incidence of all cancers in women worldwide and is the fourth most common cause of cancer mortality [2]. In general, mortality is caused by metastasis [3]. The metastatic tumour cells become more migratory and invasive through processes involving epithelial–mesenchymal transition (EMT), phenotypic plasticity and interactions with other local cells and stroma that allow escape from the primary site, survival at non-physiological sites and colonisation elsewhere [4,5]. Metastatic cells typically also become resistant to standard therapies [6]. There is, thus, a crucial need to identify new molecular targets within the process of metastasis to render it a controllable disease.

One major consequence of EMT is changes to the secretome of the tumour cells. For example, upon EMT, production of fibronectin (FN), fibrillar collagens, matrix-metalloproteinases and growth factors is increased [7,8]. These extracellular proteins contribute to the tumour microenvironment (TME), alter mechanical properties and promote tissue remodelling, all of which are associated with tumour development [9]. These processes are also driven by cancer-associated fibroblasts that develop from fibroblasts [10]. Targeting the TME is viewed in general as a therapeutic opportunity [11]. Therefore, we have become interested to consider the targeting of enzymes within the endoplasmic reticulum (ER) that are important for the folding and post-translational modification of proteins that are destined for secretion, particularly with regard to extracellular matrix (ECM) proteins.

Protein disulphide isomerase A3 (PDIA3) is of interest as a member of the PDI family of enzymes that have key roles in disulphide bond isomerisation [12]. PDIA3 is a 58 kDa, predominantly ER-resident protein that contributes to folding of Glc_2_Man_9_GlcNAc_2_-modified proteins. The substrates identified to date include a number of ECM-associated proteins [13]. PDIA3 acts as a disulphide isomerase and oxidoreductase and consists of four thioredoxin domains, the first and fourth of which are catalytically active [14]. Alterations in PDIA3 abundance have been correlated with poor prognosis in several cancers. In cervical cancer or gastric cancer PDIA3 was decreased [15,16], whereas in laryngeal cancer or aggressive prostate cancers PDIA3 was up-regulated [17,18]. In breast cancer, PDIA3 was found by immunohistochemistry to be up-regulated in aggressive primary ductal breast cancers [19]. A proteomic study of samples from Korean women to identify differentially expressed proteins between ductal carcinoma *in situ* (DCIS) or invasive ductal carcinoma (IDC) and matched normal tissue showed that PDIA3 was highly up-regulated relative to the normal tissue in both DCIS and IDC and correlated with lymph node metastasis [20]. Similar findings have been reported in other studies of breast cancer [21]. In a proteomic study of mammary glands from 21-day-old rats for proteins correlated with the cancer-preventative response of prepubertal consumption of genistein, PDIA3 was down-regulated, indicating a potential correlation of decreased levels with protection against development of breast cancer [22]. Of related interest, depletion of PDIA3 in MDA-MB-231 breast cancer cells reduced chemoresistance-associated proliferation [23]. *In vitro*, differential transcriptome and proteome analyses of the bone metastasis-prone MDA-MB-231-BO2 breast cancer cell line versus parental MDA-MB-231 cells identified PDIA3 as a mediator of bone metastasis-associated proteins [24]. PDIA3 was also shown to be involved in EGF receptor signalling in MDA-MB-468 breast cancer cells [25] and to promote growth of SUM159PT mammosphere cultures [26].

Considering the above associations of PDIA3 with breast cancer progression and its known ECM glycoprotein substrates, our goal was to investigate the possible role of PDIA3 in pro-metastatic phenotypes of human breast cancer cell lines *in vitro*, and the possible functional role of secreted proteins that depend on PDIA3 function for their post-translational modification and secretion. As a first approach to this question, we have analysed effects of pharmacological inhibitors and used conditioned media (CM) from wildtype (WT) or *Pdia3^−/−^* mouse embryonic fibroblasts (MEFs) on three breast cancer cell lines representing luminal or basal phenotypes, with regard to *in vitro* properties of cell attachment, spreading and migration that underpin metastatic cell phenotypes *in vivo.*

## Materials and methods

### Cell culture and materials

All chemicals were from Sigma unless otherwise stated and catalogue numbers are given. MDA-MB-231 [27], HCC1937 [28] and MCF-7 [29] cells were obtained from ATCC and MDA-MB-231 and MCF7 were cultured in high-glucose DMEM (D5671); HCC1937 were cultured in RPMI (R8758), each containing 10% foetal bovine serum (FBS) (F2442) in a sterile, 37°C humidified, copper-lined incubator gassed with 5% CO_2_. *Pdia3^−/−^* and WT MEFs [30], were kind gifts from Professor Michalek, University of Alberta, Canada and were grown in Fibroblast Growth Medium (FGM) (C23110, Promocell). Primary antibodies used included rabbit monoclonal anti-PDIA1(protein disulphide isomerase A1; C81H6; Cell Signaling Technology) and rabbit anti-PDIA3 (Ab137456, Abcam). Recombinant human PDIA1 (ENZ-51024) was from Enzo and recombinant human PDIA3/ERp57 (ab92937) was from Abcam. Specimens of breast carcinomas with basal (grade 3 IDC ER^−^ PGR^−^ HER2^−^) or luminal (grade 3 IDC ER^+^ PGR^+^ HER2^−^) histology from age-matched female patients were obtained as anonymous samples from the Wales Cancer Bank (www.walescancerbank.com) as sections of formalin-fixed, paraffin-embedded tumour biopsies. The study was approved under the Human Tissue Act (HTA 16/WA/0256) as WCB project number 17/020.

### Immunohistochemistry

Slides were de-waxed in Histoclear (National Diagnostics, Atlanta, U.S.A.) then re-hydrated by sequential washes in 100 and 70% ethanol, and then water. Antigen retrieval was carried out in hot 10 mM sodium citrate buffer at pH 6.0 for 20 min. Samples were quenched in 0.6% H_2_O_2_ (H1009) for 17 min and washed twice for 2 min in phosphate buffered saline (PBS). Immunohistochemistry was performed with a rabbit antibody to PDIA3 (Ab137456, Abcam) at 1:500 dilution for 30 min, followed by Vectastain Universal Elite ABC immunohistochemistry kit (with 1:50 dilution of Universal secondary antibody) and ImmPACT DAB peroxidase substrate detection reagent (all in kit PK6200, from Vector Labs, Peterborough, U.K.). Slides were then washed in cold running water for 5 min and counter-stained in Gill’s Haematoxylin (GHS216). Staining with non-immune rabbit IgG (NIO1, Sigma) as a control was included in each set of slides to assess any background diaminobenzidine tetrahydrochloride (DAB) reactivity. Images were taken under the 20× bright-field objective of a Leica DMI4000B microscope using a Leica DFC410 FX CCD camera controlled by LAS 3.7 software and exported as tif files.

### Determination of inhibitor concentrations for cell-based assays

After trypsinisation from stock culture, cells were washed three times in FGM and plated in FGM in a 24-well cell culture tray (Falcon 353226) at 2.6 × 10^4^ cells/cm^2^ (5 × 10^4^cells/well). Wells were treated in duplicate with various concentrations of either PACMA-31 (SML-0838) or 16F16 (SML-0021) or dimethyl sulphoxide (DMSO, D2650) only (8.46 mM (0.1% v/v final)) as a control. Trays were incubated in an IncuCyte ZOOM™ (Essen BioScience) ‘in incubator’ live-cell imaging system with 10× (0.3 NA) objective and 25 images per well taken automatically once an hour for 24 h. The software’s inbuilt ‘confluence-mask’ setting was used to calculate the change in ‘% cell density’ for each condition (Supplementary Figure S1). Substantial cell death was observed above 10 µM of 16F16. HCC1937 cells were too flat to be detected by the IncuCyte ZOOM™ software so the change in cell morphology was analysed manually by counting flat, attached cells compared with rounded cells. This experiment identified optimised inhibitor concentrations that were used in following experiments: for HCC1937 cells, 7.5 µM 16F16 or 0.75 µM PACMA-31; for MDA-MB-231 cells, 5 µM 16F16 or 2.5 µM PACMA-31 and for MCF-7 cells, 2 µM 16F16 or 5 µM PACMA-31.

### Measurement of breast cancer cell attachment and spreading

The inhibitor conditions above were used to treat breast cancer cells plated in FGM at 3 × 10^5^cells/cm^2^ for 24 h. Cells were then re-plated into test dishes according to the experimental design. To measure attachment and spreading, breast cancer cells were seeded on to glass coverslips in six-well trays (Falcon 353934) at 1.3 × 10^5^ cells/cm^2^ (2.5 × 10^5^ cells/well) and re-treated with the same concentration of inhibitor or equivalent volume of DMSO. After 2, 6, 12 or 24 h at 37°C, medium was gently removed and the cells washed three times in PBS, then fixed in 4% paraformaldehyde (PFA; 28906, Thermo Fisher Scientific) in PBS for 10 min. Fixed cells were permeabilised in 0.5% Triton-X 100 (T6066) for 10 min, then stained for 50 min at room temperature (RT) in a humidified chamber with phalloidin-fluorescein isothiocyanate (P5282), washed three times and mounted in VectaShield® with DAPI (H-1200, Vectorlabs). In some experiments, MDA-MB-231 cells were stained for 2 h at RT with mouse α-vinculin IgG (V4505) in 2% bovine serum albumin (F7524) in PBS, then washed and incubated with Alexa Fluor®488 conjugated α-Mouse IgG (A11001, Life Technologies). Cells were then washed and mounted as stated above. Samples were examined with a Leica SP5-AOBS confocal laser scanning microscope attached to a Leica DM I6000 inverted epifluorescence microscope with an HCX PL APO λ blue 63× 1.4 NA oil objective and Leica Application Suite AF software 2.7.3.9723. Z-stack images were taken at zoom 1 at approximately 0.25 µm z-slice thickness with an overall z-depth of 5 µm. A 100-mW 488-nm Argon laser was used to detect Alexa Fluor®488 and Phalloidin-FITC and a 50-mW 405-nm diode laser for DAPI. Two coverslips were imaged per condition per experiment with six to eight z-stack images taken per experiment such that approximately 50 cells could be scored. Images were saved as tif files, and are displayed as single channel ‘Maxintensity’ z-stack merges or Maxintensity merged red-green-blue (RGB) images. Cell outlines were defined from the single channel Maxintensity merges of phalloidin-stained cells with the ImageJ freehand outline tool, or automated threshold- > binary- > measure particles where possible, and then measured by calibrated region-of-interest (ROI) in ImageJ to obtain cell areas. Immunofluorescent images of vinculin staining were z-projected into a max-intensity stack. The resultant image was then filtered using the ‘Yen’ threshold in ImageJ (Fiji). This threshold allowed conversion into a binary image, where focal adhesions were measured using the Analyze Particles function, set to exclude on edges and to record a minimum particle size of 2 μm^2^. In parallel, cell areas were measured as described above. For quantification of cell attachment, the number of nuclei was counted manually over 20 random 63× fields (at 2 and 6 h time points) or seven random 40× fields (at 12 and 24 h) per experiment.

### Effects of CM from *Pdia3^−/−^* or WT MEFs on breast cancer cell attachment and spreading

CM was prepared by plating WT or *Pdia3^−/−^* MEF, each at 2 × 10^4^ cells/cm^2^ in serum-free FGM containing 300 µM l-ascorbic acid (A4403) for 48 h at 37°C. CM was harvested and passed through a 0.22-µm filter (SLGP033RS, Millex-GP) to ensure sterility and remove cell debris. Breast cancer cells were seeded on glass coverslips at 2.8 × 10^4^ cells/cm^2^ in either CM or fresh FGM. After 2 h at 37°C, medium was removed, cells washed three times in PBS to remove non-adherent cells and fixed in 4% PFA in PBS for 10 min. Fixed cells were processed for F-actin staining and image analysis as described above.

### ‘Scratch wound closure’ migration assay

Cells were plated and treated with inhibitors or DMSO only as the solvent control (8.46 mM, (0.1% (v/v)) as described above, then MDA-MB-231 or HCC1937 cells were seeded in FGM at 1.25 × 10^5^ cells/cm^2^ into IncuCyte® ImageLock 96-well trays (4379), as described for the inhibitor concentration experiment, and incubated with the same concentration of inhibitor or equivalent volume of DMSO (corresponding to 8.46 mM (0.1% v/v final) DMSO) at 37°C overnight to form monolayers. MCF-7 cells were found to form irregular layers in FGM, and so were seeded as above at 1.6 × 10^5^ cells/cm^2^ in DMEM with 10% FBS containing either inhibitor or equivalent volume of DMSO and allowed to attach for 24 h. In experiments where MDA-MB-231 cells were treated with CM, cells were seeded as above in FGM and left overnight to attach. Then wells were washed twice in PBS and then CM from WT or *Pdia3^−/−^* MEF added. All wells were ‘scratched’ with the 96-pin WoundMaker™ (Essen). For MCF-7 cells, medium was replaced with fresh DMEM with 10% FBS containing inhibitor or DMSO and imaging carried out hourly for 24 h only using the IncuCyte ZOOM™. For other cells, wells were imaged every hour over 48 h. Experiments involving incubation with CM were imaged for 24 h only. Change in ‘relative wound density (RWD)’ over the time period was then calculated for each condition from the images by the standard IncuCyte masking procedure.

### HCC1937 cell attachment and spreading on ECM

HCC1937 cells were seeded at 2.6 × 10^5^ cells/cm^2^ (5 × 10^5^ cells/well) on to glass coverslips in six-well trays and treated with the optimised inhibitor concentrations or equivalent volume of DMSO. After 48 h at 37°C, the medium was gently removed, cells were washed three times in PBS and lysed with ammonium hydroxide (44273, Honeywell Fluka) for 5 min with rocking [31]. Cell lysate was removed by washing five times in large volumes of sterile dH_2_O, leaving the coverslips with isolated ECM that were kept in sterile PBS. HCC1937 cells grown under normal conditions (naïve cells) were trypsinised and washed twice in FGM and plated at 1.3 × 10^5^ cells/cm^2^ (2.5 × 10^5^ cells/well) in FGM on to ECM and incubated for 1 h at 37°C. Medium was then gently removed, cells washed in PBS, fixed and stained for F-actin as described above. Samples were imaged with the Leica DMI4000B as detailed above. XY images were taken from three or four random locations on the coverslips and saved as tif files. Cell outlines were defined using ImageJ threshold- > binary- > measure particles or freehand outline (to measure the few that could not be detected automatically) and areas quantified by ROI analysis in ImageJ as above. A minimum of 150 cells was measured per condition per experiment for a total of approximately 400 cell area measurements per condition. Attachment was measured by counting the number of nuclei across seven random 40× fields per experiment.

### Sample preparation for sodium dodecyl sulphate/polyacrylamide gel electrophoresis and Western blotting

Breast cancer cell lines grown under standard conditions were washed three times in FGM and seeded at 4 × 10^4^ cells/cm^2^ in 2 ml of FGM containing optimised concentrations of PACMA-31, 16F16 or equivalent volume of DMSO in a p60 cell culture dish (Falcon 353002). After 6 or 24 h at 37°C, medium was removed and centrifuged at 400×***g*** for 5 min to remove cell debris. Each supernatant was passed through a sterile 0.22-µm filter, mixed with 1× protease inhibitor cocktail (A32953, Thermo Fisher Scientific) and chilled on ice. Heparin-binding proteins were isolated by incubation with 25 µl Affi-gel® heparin-agarose beads (153-6173, Bio-Rad) with rotation at 4°C for 1 h. Beads were washed three times in TBS containing 2 mM CaCl_2_, pelleted again and bound proteins eluted by mixing 1:1 with sodium dodecyl sulphate/polyacrylamide gel electrophoresis (SDS/PAGE) sample-buffer containing 100 mM DTT and boiling for 5 min. Cells were rinsed in PBS and whole cell extracts collected separately in 150 µl SDS/PAGE sample buffer containing 100 mM DTT using a cell scraper. MEFs at ∼80% confluence were also lysed by this method. Proteins were resolved on 10% polyacrylamide gels under reducing conditions, transferred to polyvinylidene fluoride (PVDF) membrane (ISEQ00010, Merck) by semi-dry transfer and membranes probed for FN or glyceraldehyde 3-phosphate dehydrogenase (GAPDH) using α-FN rabbit monoclonal (F3648), rabbit monoclonal anti-PDIA1 (C81H6; Cell Signaling Technology) or rabbit anti-PDIA3 (Ab137456, Abcam) with horseradish peroxidase (HRP)-conjugated α-Rabbit IgG (926-80011, Li-COR), or α-GAPDH mouse monoclonal (ab9484, Invitrogen) or mouse α-vinculin IgG (V4505), and HRP-conjugated α-Mouse IgG (926-80010, Li-COR), respectively. Antibody binding was detected using Amersham ECL Western blot detection reagent (RPN2209, Amersham) and imaged digitally in a Syngene G:BOX Chemi XRQ. Band quantification was performed with Syngene GeneTools software.

### Replication of experiments and presentation of graphs

All cell-based experiments were performed three times independently, with technical replicates and controls in each experiment, unless stated otherwise in the figure legend. Graphs describing the scratch wound metrics were plotted using the ggplot2 R extension. All other graphs were plotted using GraphPad Prism.

### Statistical analysis

Data were plotted as a frequency histogram using R with basic descriptors such as Shapiro–Wilk and skewness/kurtosis to determine normality. Data were then analysed using two-way ANOVA with Tukey’s multiple comparisons post-hoc test through GraphPad Prism or R, and displayed as median ± standard deviation (S.D.) unless otherwise stated. Because the majority of data distributions were non-parametric, the ANOVA/Tukey’s *P*-values were verified using the inbuilt GraphPad Prism 7.0 Kruskal–Wallis non-parametric multiple comparisons test with Dunn’s multiple corrections. Where possible, tests were repeated on normalised data (log transformed).

## Results

### Presence of PDIA3 in human breast tumours and breast cancer cell lines

**Figure 1:**
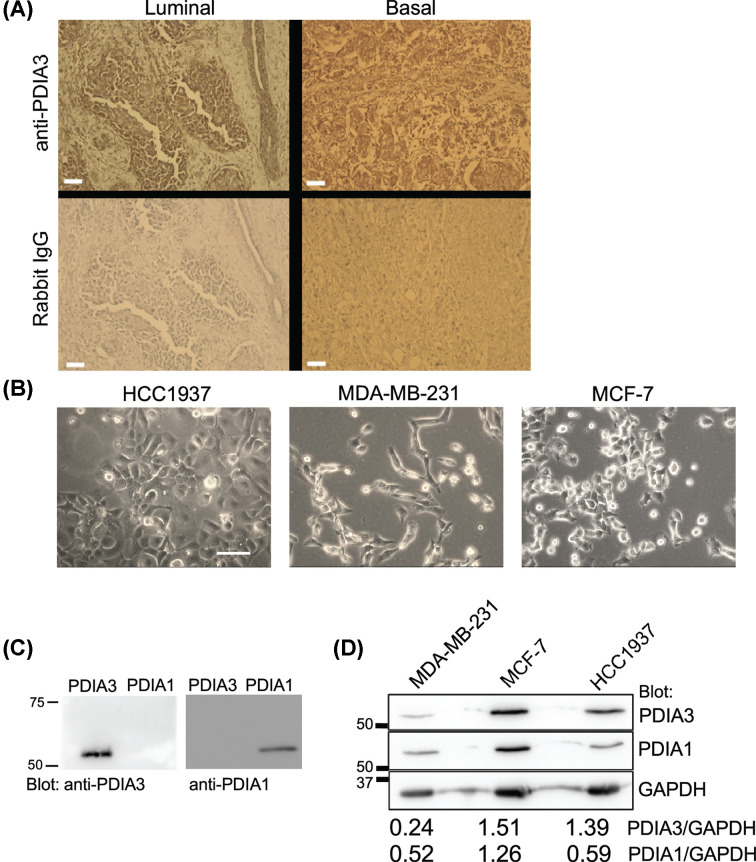
Presence of PDIA3 in human breast cancer and breast cancer cell lines (**A**) Immunohistochemistry for PDIA3 in human luminal and basal IDCs. Bar = 50 μm. (**B**) Phase contrast images of HCC1937, MDA-MB-231 or MCF-7 breast cancer cells in culture. Bar = 100 µm. (**C**) Specificity of the antibodies against PDIA1 and PDIA3, respectively. Western blot of the recombinant human proteins (50 ng per lane) was probed with each antibody. (**D**) Western blot of cell lysates from each cell line, showing presence of PDIA3 and the related PDIA1. Numbers refer to quantification versus GAPDH from this blot.

The presence of PDIA3 was investigated in individual samples of primary human luminal and basal breast tumours by immunohistochemistry. In the luminal phenotype tumour, PDIA3 was present in the epithelial glandular structures and was low or absent within the stroma. In the basal-phenotype tumour, in which glandular structures are lost, PDIA3 appeared uniformly distributed (Figure 1A). Next, human breast cancer cell lines representing luminal and basal phenotypes were examined for PDIA3 and the related family member PDIA1 (33.7% sequence identity with 8% gaps for the human proteins) by immunoblotting. As shown in Figure 1B, HCC1937 cells (derived from a primary basal tumour [27]) form colonies of closely packed cells. MDA-MB-231 cells (derived from the pleural effusion of a basal tumour and have undergone EMT [28]) grow as single cells with a migratory phenotype. MCF-7 cells (derived from the pleural effusion of a luminal tumour [29]) form close-packed colonies of cells. The antibodies chosen for this study showed specificity for PDIA1 or PDIA3, when tested against the respective human recombinant proteins (Figure 1C). All the cell lines contained both PDIA3 and PDIA1 (Figure 1D). When quantified by ratioing to the housekeeping protein, GAPDH, HCC1937 and MCF-7 appeared to have similar abundance of PDIA3 whereas MDA-MB-231 cells had less PDIA3 (Figure 1D).

### Morphological responses of breast cancer cells to pharmacological inhibitors of protein disulphide isomerases

As an initial approach, two pharmacological inhibitors were used comparatively to implicate PDIA3 in *in vitro* behaviours of the three human breast cancer cell lines. No selective PDIA3 inhibitors are available at this time. PACMA-31 inhibits PDIA1 more strongly than PDIA3 or PDIA6 [32,33], whereas 16F16 inhibits PDIA1 and PDIA3 [34]. The experiments were carried out in a serum-free medium (FGM) to avoid possible artefacts or variability due to serum addition, and to align the results with following experiments which used FGM after medium-conditioning by fibroblasts. Pilot experiments tested a wide range of concentrations of each compound on each cell line to determine threshold concentrations at which cells started to detach. Live-cell imaging experiments with the IncuCyte system were then used to establish the concentration dependence of each cell line for morphological alterations from spread to rounded shapes of adherent cells (Supplementary Figure S1). From the dose–response plots, concentrations of each compound were chosen for the following experiments. The chosen concentration of 16F16 used on MCF-7 cells was effective to reduce the abundance of the known PDIA3 target, FN [35] (Supplementary Figure S1J). Production of FN by MDA-MB-231 cells and HCC1937 cells was too limited to provide clear results (Supplementary Figure S1J and data not shown, respectively).

### Effects of the pharmacological inhibitors on breast cancer cell morphology and F-actin

**Figure 2:**
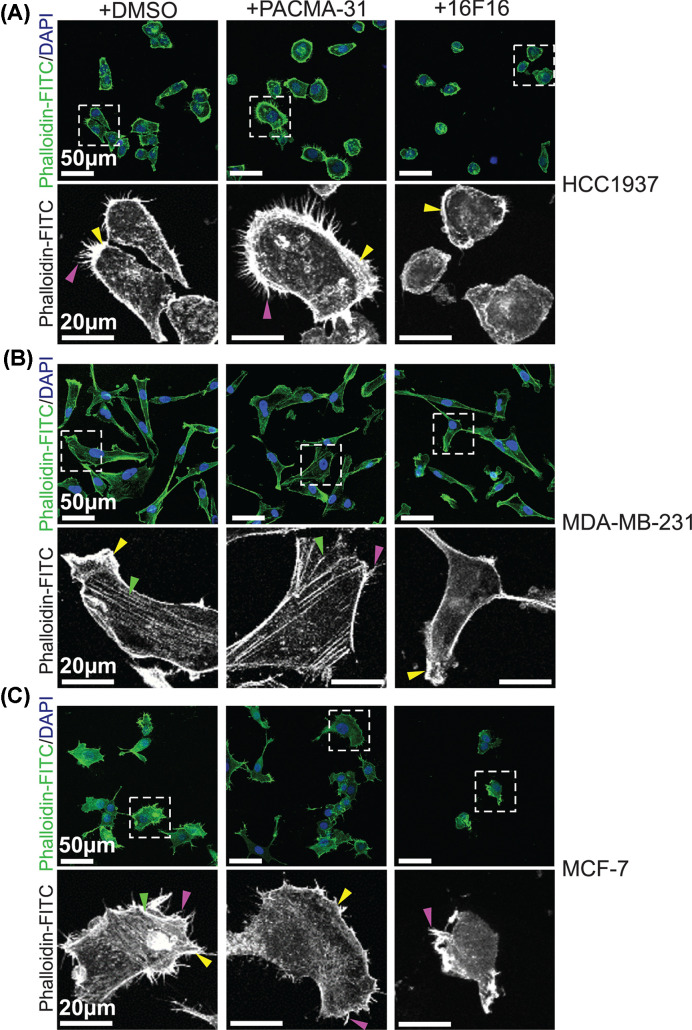
Morphological responses of breast cancer cells to pharmacological inhibitors of protein disulphide isomerases Immunofluorescence images of each cell line pre-treated as indicated, plated for 12 h in continued presence of each inhibitor or DMSO as solvent control, then processed for staining with phalloidin-FITC to visualise F-actin. (**A**) HCC1937, (**B**) MDA-MB-231 and (**C**) MCF-7 cells. The bottom row of each panel shows enlargements of the boxed areas. Arrows: yellow, lamellipodia, Green, stress fibres, Purple, finger-like protrusions. Images are representative of three independent experiments.

Breast cancer cells form various F-actin-containing structures when on rigid 2D surfaces, such as finger-like protrusions (including filopodia), stress fibres and lamellipodia [36,37]. Using the optimised concentrations of the inhibitors, we characterised effects on cell morphology and F-actin organisation over time by phalloidin staining and quantification of cell areas and cell number. At the 12 h time point, cells had adhered and appeared maximally spread, but remained clearly subconfluent. Representative images of this time point are displayed in Figure 2.

Control, DMSO-treated HCC1937 cells attached and spread in close apposition to each other with epithelial-like morphologies with bright, cell-boundary F-actin characteristic of lamellipodia (Figure 2A, yellow arrows). Most cells also formed finger-like protrusions at their edges (pink arrows). The morphologies of PACMA-31-treated HCC1937 cells were indistinguishable from the controls (Figure 2A). In contrast, HCC1937 cells treated with 16F16 appeared less spread and more rounded with frequent lamellipodia and minimal finger-like protrusions (Figure 2A). F-actin stress fibres were not detected under any condition.

As expected, control MDA-MB-231 cells had mesenchymal-like morphologies with stress fibres (Figure 2B, green arrows), lamellipodia and occasional finger-like protrusions. After PACMA-31 treatment, patterns of F-actin remained very similar to the control condition, whereas most 16F16-treated MDA-MB-231 cells showed a loss of stress fibres and finger-like protrusions yet retained cortical F-actin bundles (Figure 2B).

Control MCF-7 cells had epithelial-like morphologies with occasional stress fibres. Most cells had lamellipodia and extensive finger-like protrusions at free edges. Cells treated with PACMA-31 retained very similar morphologies, whereas cells treated with 16F16 appeared less spread and had lost most F-actin structures with only occasional cells having finger-like protrusions (Figure 2C).

### Effects of the pharmacological inhibitors on cell area and cell attachment

**Figure 3:**
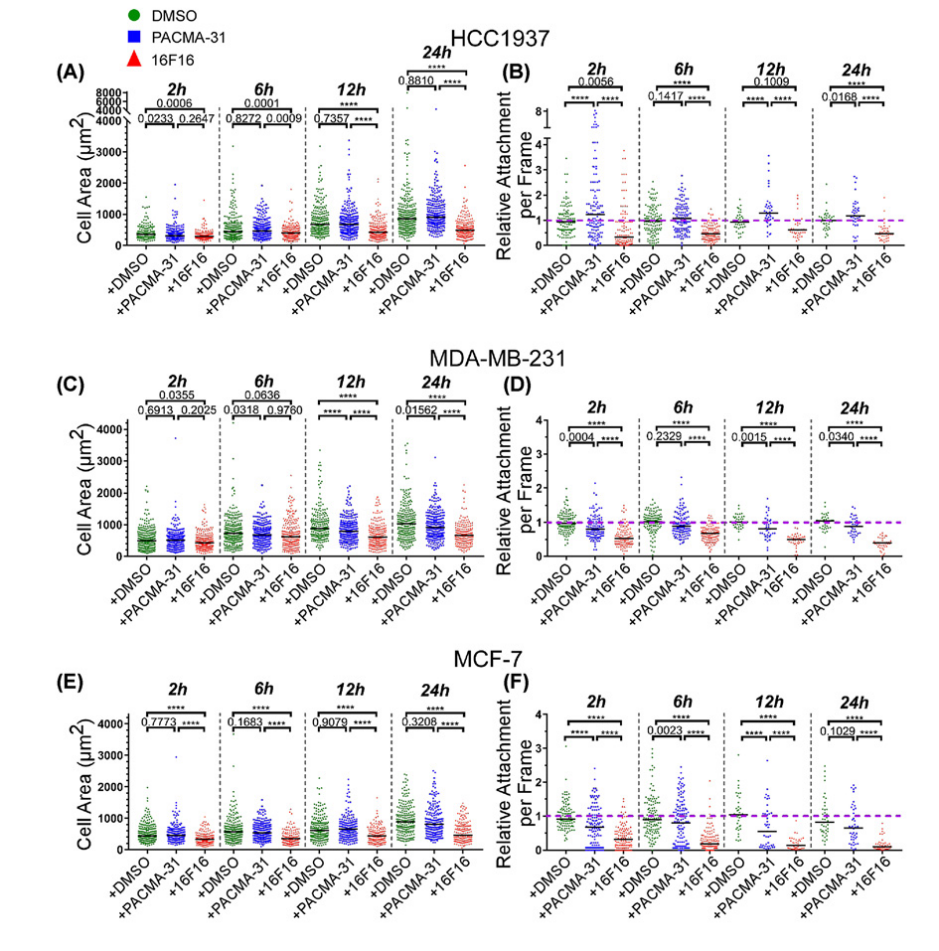
Effects of pharmacological inhibitors on breast cancer cell spreading and attachment (**A,C,E**) Quantification of cell areas of HCC1937 (A), MDA-MB-231 (C), or MCF-7 (E) cells. (**B,D,F**) Number of nuclei per microscope field for HCC1937 (B), MDA-MB-231 (D) or MCF-7 (F) cells under the indicated conditions. Purple dashed lines indicate a relative attachment of 1 (i.e. no change from the control). In each dot plot the horizontal bar indicates the median. All data analysed by two-way ANOVA with Tukey’s multiple comparison. Actual *P*-values are shown except where **** = <0.0001. Data from three independent experiments, with a minimum of 150 cells measured per condition across the three experiments.

To quantify these results, the cell areas and numbers of attached cells were measured for each cell line at time points from 2 to 24 h. At least 150 cells were measured across three independent experiments for each cell line at each time point. With regard to cell areas, control (DMSO-treated) HCC1937 cells had spread to a median area of 400 µm^2^ at 2 h and the areas increased to a median of 1000 µm^2^ by 24 h (Figure 3A). PACMA-31-treated HCC1937 cells had very similar areas at all time points, whereas 16F16-treated HCC1937 cells were significantly smaller than control cells at all time points, with a median area of only 500 µm^2^ by 24 h. 16F16-treated HCC1937 cells also had significantly reduced areas compared with PACMA-31-treated cells at the 6, 12 and 24 h time points (Figure 3A). In terms of cell numbers, the PACMA-31-treated HCC1937 cells attached in larger numbers than control cells at 2 and 12 h, but cell numbers were very similar at 6 and 24 h. HCC1937 cells treated with 16F16 had significantly reduced attachment relative to controls at all time points, and also attached less than PACMA-31 treated cells (Figure 3B). Thus, attachment and spreading of HCC1937 cells was decreased specifically by the 16F16 inhibitor.

Control and PACMA-31-treated MDA-MB-231 cells had very similar cell areas at all time-points, having a median area of 500 µm^2^ at 2 h and increasing to a median area of 1000 µm^2^ by 24 h. Only at the 12 h time-point was there a minor but significant reduction in cell area of PACMA-31-treated cells. In contrast, 16F16-treated MDA-MB-231 cells had significantly smaller areas than control or PACMA-31-treated cells at all time-points, reaching a median area of 600 µm^2^ by 24 h (Figure 3C). Fewer PACMA-31-treated MDA-MB-231 cells attached than control cells at 2 and 12 h, but cell numbers were closely similar at 6 and 24 h. 16F16-treated MDA-MB-231 cells had significantly reduced attachment compared with control cells at all time points and also attached less than PACMA-31-treated cells (Figure 3D). Thus, attachment and spreading of MDA-MB-231 cells was consistently reduced by the 16F16 inhibitor.

Control and PACMA-31-treated MCF-7 cells also had very similar cell areas at all time points, starting from a median area of 400 µm^2^ at 2 h, and increasing to a median of 900 µm^2^ by 24 h. In contrast, 16F16-treated MCF-7 had significantly smaller areas than control cells at all time points, reaching a median area of 400 µm^2^ at 24 h (Figure 3E). In terms of cell numbers, fewer PACMA-31-treated MCF-7 cells attached than control cells at 2, 6 and 12 h, but cell numbers were very similar at 24 h. 16F16-treated MCF-7 cells had significantly reduced attachment at all time points, and also attached less than PACMA-31 treated cells (Figure 3F). Thus, 16F16 inhibitor specifically reduced the adhesion of MCF-7 cells. These results demonstrated that all the breast cancer cell lines were strongly reduced in spreading and attachment by 16F16 treatment, whereas the response to PACMA-31 was more variable and minor. Under all conditions, there was no indication of nuclear fragmentation in the attached cells.

### Effects of the pharmacological inhibitors on migration of breast cancer cells

**Figure 4:**
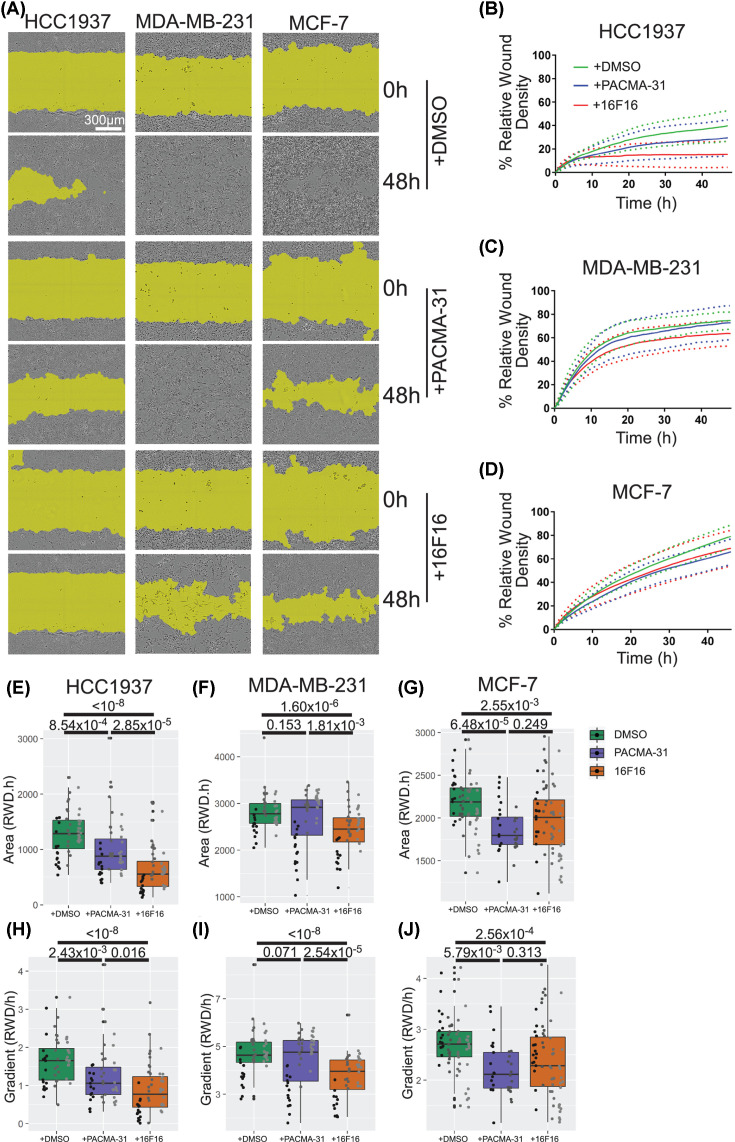
Effects of pharmacological inhibitors on ‘scratch wound’ migration of breast cancer cells (**A**) Representative images of the ‘scratch-wound’ mask (yellow) overlaid on phase contrast images from the IncuCyte® system for each cell line at the start (0 h) or end (48 h) of the time courses. (**B**–**D**) Quantification of cell migration presented as change in % RWD for each cell line. For each condition, the plots show the mean (solid line) *SD* (dotted line). For HCC1937 and MDA-MB-231 cell, data are from three experiments in FGM. For MCF-7 cells, data are from four experiments (DMSO and 16F16 conditions) or two experiments (PACMA-31 condition) in DMEM containing 10% FBS. (**E–J**) Analysis of data from (B–D) as box plots of ‘Area under the Curve’ (E–G) or as ‘Rate of Initial Wound Closure’: the change in % RWD per hour over the first 5 h (H–J). In (E–J), each dot represents the value calculated for each metric from each well in each independent experiment. Within each box plot, each vertical column of dots represents a single experiment. Data were analysed by two-way ANOVA with Tukey’s post hoc analysis in R and plotted using ggplot2.

Because the F-actin structures associated with cell motility were decreased in cells treated with 16F16, we examined the migratory capacity of the breast cancer cell lines by a live cell ‘scratch-wound’ assay in which the cells were imaged automatically once an hour for 48 h after ‘scratching’. The images are processed by application of a mask according to automated criteria of the IncuCyte software, so that % cell density over time can be tracked. An example of the general accuracy of the process is given in Supplementary Figure S2. Representative images of the ‘scratch-wound’ closure captured by the software are shown in Figure 4A. From replicate experiments, rates of scratch closure were quantified as ‘RWD’ over the time course (Figure 4B–D). HCC1937 cells rarely sealed the wound by 48 h under control, DMSO-treated conditions, whereas MDA-MB-231 and MCF-7 cells achieved relatively complete wound closure (∼70% RWD). PACMA-31 treatment impaired the ability of HCC1937 and MCF-7 cells to seal a wound compared with the control condition, whereas motility of MDA-MB-231 cells was unaffected. 16F16 treatment reduced wound closure by HCC1937 cells compared with the control and PACMA-31 conditions, and blocked wound closure by MDA-MB-231 cells. 16F16-treated MCF-7 cells had reduced wound closure in comparison with control cells, but not compared with PACMA-31-treated cells (Figure 4B–D).

These time courses were analysed further by two descriptive metrics to compare the shapes of the curves (Figure 4E–J). The initial gradient provides a metric for the initial rate of cell migration. The area under the curve (AUC) quantifies the extent of wound closure over the full time course. The AUC metric showed that PACMA-31-treated HCC1937 cells had significantly reduced ‘wound’ closure compared with control cells, and 16F16-treated HCC1937 cells had significantly reduced closure compared with both control and PACMA-31-treated cells (Figure 4E). ‘Wound’ closure by PACMA-31-treated MDA-MB-231 cells was very similar to the control, whereas 16F16-treated MDA-MB-231 cells had significantly reduced closure compared with either control or PACMA-31-treated cells (Figure 4F). Compared with control MCF7 cells, PACMA-31- and 16F16-treated MCF-7 cells each showed significantly reduced closure (Figure 4G). The same differences between the conditions for each cell line were also apparent from the calculated initial gradients (Figure 4H–J).

Overall, 16F16 treatment reduced initial rates of closure and overall ‘scratch’ closure for all the cell lines, whereas PACMA-31 reduced the migration of HCC1937 and MCF7 cells to a lesser extent. For HCC1937 cells, 16F16 clearly had a stronger effect than PACMA-31. None of the conditions led to overt cell detachment or death (Figure 4A).

### Effects of CM from *Pdia3^−/−^* and WT MEFs on morphologies of breast cancer cell lines

**Figure 5:**
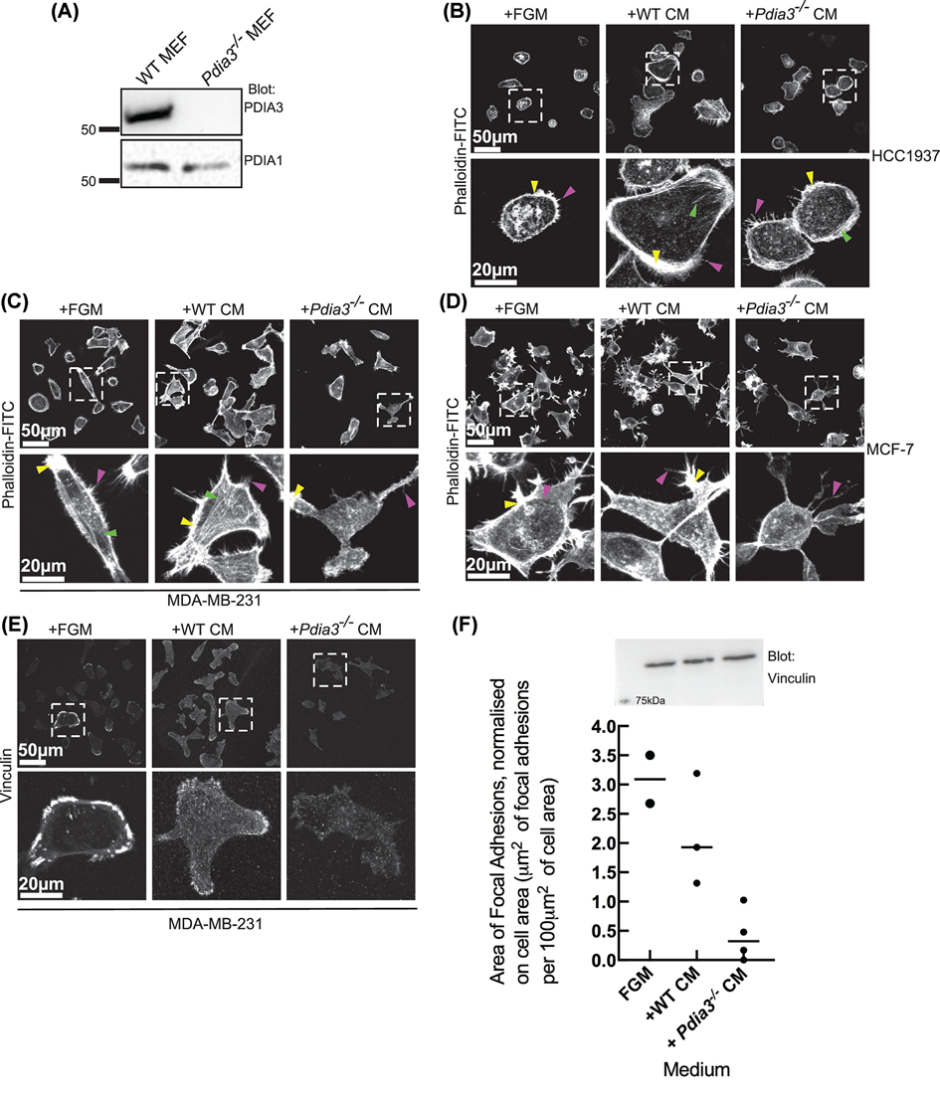
Effects of CM from WT or *Pdia3^−/−^* MEFs on morphology of breast cancer cell lines (**A**) Western blot of WT or *Pdia3*^−/−^ MEF cell lysates showing absence of PDIA3 in the *Pdia3*^−/−^ MEF. (**B–D**) Representative F-actin organisation in the three cell lines after adhesion for 2 h with the indicated medium. The bottom row of each panel shows enlargements of boxed areas. Arrows: Yellow, lamellipodia, Green, stress fibres, Purple, ‘finger-like’ protrusions. (**E**) Vinculin localisation in MDA-MB-231 cells under the same conditions as in (C). (**F**) Quantification of area occupied by focal adhesions from vinculin staining of adherent cells as in (E). Data are presented normalised per 100 μm^2^ of cell area. Horizontal lines indicate the median. Data are from at least 35 cells/condition from three random images. Upper panel shows vinculin immunoblot of whole cell extracts under the different conditions (single experiment).

Tumour cell interactions with fibroblasts have important roles in driving tumour progression [10] and PDIA1 and PDIA3 have known substrates that are destined for secretion [13,35]. As a separate approach to investigate the functional role of PDIA3, we tested how CM produced from WT or *Pdia3^−/−^* MEFs, grown in serum-free FGM, would affect attachment or spreading of the breast cancer cell lines. First, whole cell lysates of *Pdia3^−/−^* or WT-MEF were immunoblotted for PDIA3 to verify loss of PDIA3 in the gene-knockout MEF, whereas PDIA1 is maintained (Figure 5A). Next, effects of each CM on initial cell spreading and attachment of the breast cancer cell lines was assessed by plating the breast cancer cells into either CM or fresh FGM for 2 h, then fixing and staining for F-actin. This early time point was chosen to focus the experiment on initial cell attachment and spreading, when production of endogenous ECM by the breast cancer cells is very limited.

HCC1937-cells attached and spread in fresh FGM with clear peripheral F-actin structures including finger-like protrusions (Figure 5B, pink arrows) and lamellipodia (Figure 5B, yellow arrows). Exposure to CM from WT-MEF (WT-CM) resulted in formation of F-actin stress fibres (Figure 5B, green arrows) and enhanced finger-like protrusions and lamellipodia. Cells exposed to CM from *Pdia3^−/−^* MEF (KO-CM) had very similar patterns of F-actin to cells grown in fresh FGM (Figure 5B).

Compared with MDA-MB-231 cells plated in FGM, MDA-MB-231 cells plated in WT-CM exhibited enhanced, brighter F-actin structures (Figure 5C). Cells plated in KO-CM had more irregular cell shapes with fewer stress fibres (green arrows) and finger-like protrusions (pink arrows) (Figure 5C).

Under all the conditions, MCF-7 cells had highly variable and irregular shapes, so any morphological responses to either CM were less clear. MCF-7 cells in fresh FGM spread well and formed F-actin-rich lamellipodia (yellow arrows) and extensive finger-like protrusions (pink arrows) as expected (Figure 5D), whereas MCF-7 cells plated in WT-CM appeared to have more projections at cell edges. Compared with these conditions, MCF-7 cells plated in KO-CM had reduced lamellipodia and any finger-like protrusions were often long and curved (Figure 5D).

Because MDA-MB-231 had the strongest difference in F-actin organisation upon exposure to KO*-*CM, the localisation of vinculin, a component of focal adhesions, was also examined in this cell line under the three experimental conditions. In FGM, MDA-MB-231 cells formed distinct peripheral focal adhesions. Cells plated in WT-CM also formed focal adhesions, but these were smaller and present under cell bodies and less obviously clustered at peripheries. Cells plated in KO-CM had a marked reduction in focal adhesions compared with the cells plated in WT-CM or FGM (Figure 5E). Quantified image analysis confirmed that the area occupied by focal adhesions, when normalised per 100 μm^2^ of cell area, was reduced in cells plated in KO-CM, whereas total vinculin abundance was maintained (Figure 5F).

Thus, CM produced by WT-MEF enhanced F-actin organisation in all three cell lines relative to fresh FGM, whereas CM produced by *Pdia3^−/−^* MEF had little effect. Also, vinculin localisation in focal adhesions of MDA-MB-231 cells was reduced in response to KO-CM compared with WT-CM or FGM. These data implicate that there are PDIA3-dependent secreted products of fibroblasts that provide an environment favourable for breast cancer cell spreading and F-actin and vinculin organisation.

### Effects of CM from *Pdia3^−/−^* and WT MEFs on cell spreading and attachment of breast cancer cell lines

**Figure 6:**
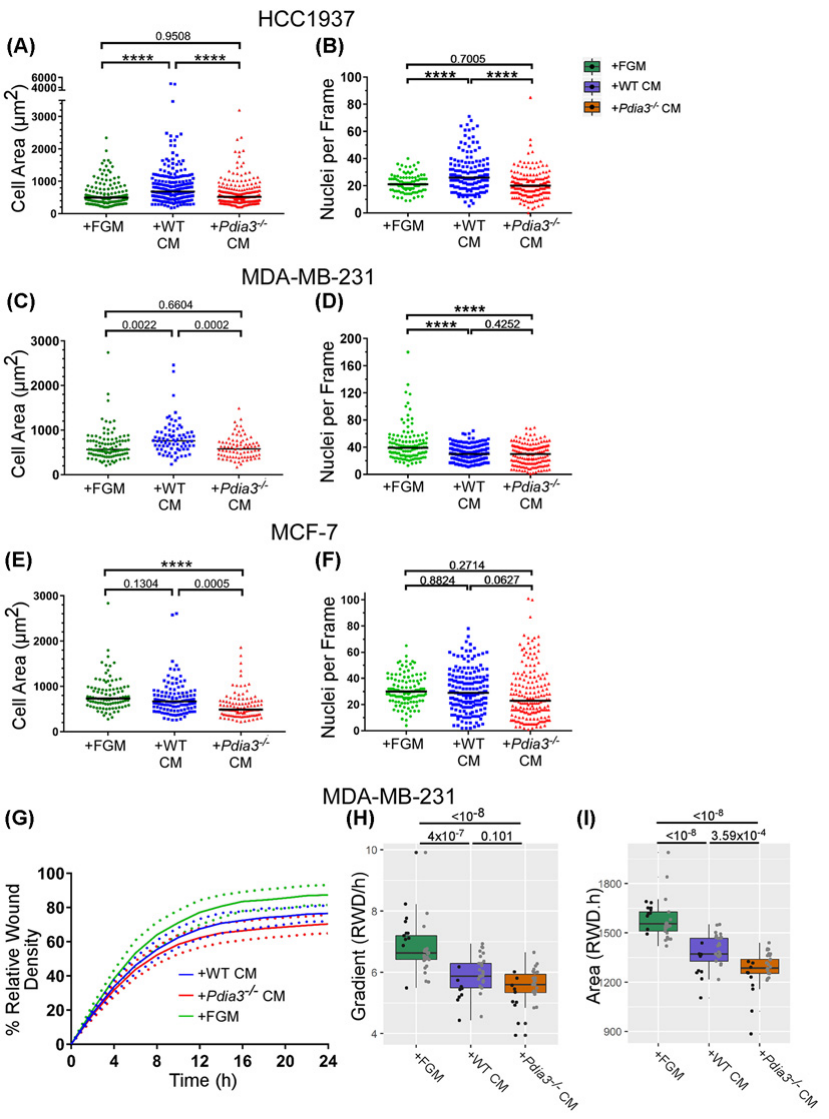
Effects of CM from WT or *Pdia3^−/−^* MEFs on attachment and spreading of breast cancer cell lines (**A,C,E**) Cell areas for each cell line (HCC1937 (A), MDA-MB-231 (C) or MCF-7 (E) under the same conditions as in Figure 5. (**B,D,F**) Cell attachment scored from nuclei per 63x microscope frame. In each dot plot, the horizontal line shows the median. (**G**) Data from time courses as change in % RWD for MDA-MB-231 cells plated for 24 h under the indicated conditions. For each condition, the mean (solid line) *SD* (dotted line) are shown. Data are from two experiments. (**H,I**) Analyses of the time course data, as box plots of ‘AUC’ (**H**), or ‘Rate of Initial Wound Closure’ (I). In (H,I), each dot represents the value calculated for each metric from each well in each independent experiment. Within each box plot, each vertical column represents a single independent experiment. All data analysed by two-way ANOVA with Tukey’s post hoc analysis in R and plotted using ggplot2.

The above observations of cell morphologies were substantiated by quantification of spread cell areas and numbers of attached cells. HCC1937 in FGM cells had a median cell area of ∼500 µm^2^ and cell area increased in response to WT-CM to ∼700 µm^2^ but was unaltered by KO-CM. The cell areas achieved after plating in KO-CM were significantly smaller that in WT-CM (Figure 6A). WT-CM also increased the number of attached cells whereas similar numbers of cells attached in FGM or KO-CM. Thus, attached cell numbers were significantly reduced in KO-CM relative to WT-CM (Figure 6B).

MDA-MB-231 cells in fresh FGM had a median area of ∼600 µm^2^. In WT-CM, spreading of cells increased to ∼800 µm^2^. KO-CM did not promote spreading relative to FGM and the cell areas achieved in KO-CM were significantly smaller than those of cells plated in WT-CM (Figure 6C). For this cell line, cell attachment was highest in fresh FGM (Figure 6D).

MCF-7 cells in fresh FGM had a median area of ∼700 µm^2^. Cells plated in WT-CM had similar areas, whereas cells plated in KO-CM were significantly smaller, with a median area of ∼500 µm^2^ (Figure 6E). Attached cell numbers were similar in all three conditions (Figure 6F).

Overall, the results obtained were cell-line dependent. CM from *Pdia3^−/−^* MEF had a reduced capacity relative to WT-CM to support cell spreading of all the cell lines, and a reduced capacity to support attachment of the basal-like cell line HCC1937.

Because MDA-MB-231 cells showed a clear alteration in spreading in *Pdia3^−/−^* CM compared with WT-CM, and were the most migratory cells, the responses of these cells to CM were also examined in the ‘scratch wound’ closure assay. A 24-h time course was chosen because, in pilot experiments, CM older than 72 h (48 h to prepare CM + 24 h for the scratch assay) was found not to support cell growth. MDA-MB-231 cells in FGM sealed a wound effectively (∼85% RWD at 24 h). Cells in WT-CM had a reduced ability to seal the wound (∼75% RWD), and wound closure was further reduced in KO-CM (∼70% RWD) (Figure 6G). Analysis by two descriptive metrics to compare the shapes of the curves (Figure 6H,I) showed that the initial rate of migration of cells in WT-CM or KO-CM was significantly reduced by a similar amount compared with cells in fresh FGM (Figure 6H). However, cells in KO-CM were clearly less able to close the wound within the time period (Figure 6I). The data implicate that there are PDIA3-dependent secreted products of fibroblasts that support overall wound closure but are not overtly active in the initial rate of wound closure.

### Effects of ECM produced by inhibitor-treated HCC1937 cells on attachment and spreading of naïve HCC1937 cells

In view that secreted products of WT MEFs were found to promote breast cancer cell spreading and motility and the CM of *Pdia3^−/−^* MEF had reduced activity, we next considered the effect of the pharmacological inhibitors on autocrine, secreted proteins. HCC1937 cells were used for this experiment because they showed clear differences in cell attachment and area in response to either 16F16 or KO-CM. HCC1937 cells were grown for 48 h under the optimised inhibitor conditions or control conditions, and then the assembled ECM was isolated by established procedures [31]. Fresh ‘naïve’ HCC1937 cells in FGM were plated onto an ECM for 1 h. An early time point was used because initial cell attachment phenotypes were the focus of this experiment, and to minimise the interference from ECM production by the newly plated cells (which would become an issue at later times).

**Figure 7:**
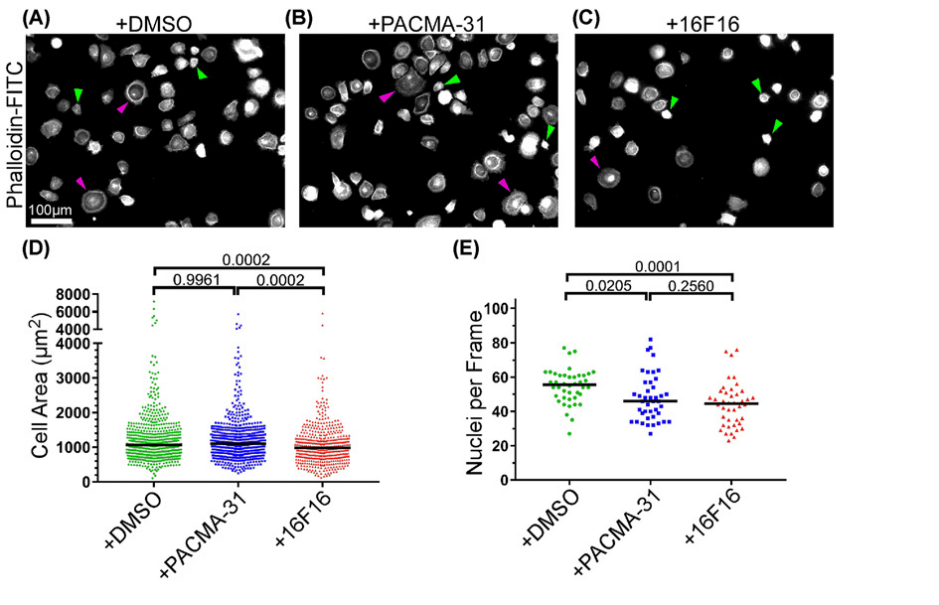
Effects of pharmacological inhibitors on support of cell spreading and attachment of naïve HCC1937 cells by HCC1937 ECM (**A**–**C**) Morphologies of HCC1937 cells plated for 1 h on to isolated ECM, prepared from HCC1937 cells treated as indicated. Green arrows indicate small, rounded cells. Pink arrows indicate well-spread cells. (**D**) Dot plots of cell areas. (**E**) Dot plots of numbers of attached cells. In (D,E), horizontal lines indicate the median and at least 500 cells were scored for each condition across three independent experiments.

ECM from control HCC1937 cells supported rapid attachment and extensive spreading (Figure 7, pink arrows) of fresh HCC1937 cells. Some smaller, rounded cells were also apparent (Figure 7, green arrows). ECM from PACMA-31-treated cells supported very similar cell spreading (Figure 7A). Cells plated on ECM from 16F16-treated HCC1937 cells appeared less spread and small round cells were frequently observed (Figure 7C). Quantification of cell areas from three independent experiments showed that the ECM produced under control conditions supported HCC cell spreading to a median area of 1000 µm^2^, with most cells between 500 and 1600 µm^2^ (Figure 7D). However, there was noticeable variation in cell areas, with the distribution also including some much larger cells (>2500 µm^2^) (Figure 7D). Cells on ECM from PACMA-31-treated cells had similar areas, whereas cells plated on ECM from 16F16-treated cells had significantly smaller areas (median ∼900 µm^2^), less variability of cell areas (range 400–1200 µm^2^) and few very large cells. Cell attachment to ECM produced by 16F16-treated cells was reduced compared with the DMSO condition but not compared with the PACMA-31 condition (Figure 7E). This was because ECM produced by PACMA-31-treated cells caused an intermediate reduction in cell attachment. Overall, ECM of 16F16-treated cells had reduced activity to support cell attachment and spreading compared with the other conditions.

## Discussion

Here, we implicate a functional role of protein disulphide isomerases in facilitating adhesion and motility of human breast cancer cell lines, with regard to fundamental activities of cell attachment, spreading, organisation of motility-associated F-actin structures, and cell migration. The pharmacological inhibitors tested both act to inhibit PDIA3, and a potential PDIA3-dependent function in extracellular interactions between fibroblasts and cancer cells was investigated using CM from WT or *Pdia3^−/−^* MEF. Treatment with CM from *Pdia3^−/−^* MEF reduced initial spreading of cells and formation of migratory F-actin structures relative to WT-CM. Using MDA-MB-231 cells, CM from *Pdia3^−/−^* MEF was found to have reduced activity to stimulate either vinculin organisation or cell migration into a ‘scratch-wound’ compared with FGM. Finally, we showed that ECM of HCC1937 cells produced under pharmacological inhibition by 16F16 was less supportive than control ECM for initial attachment and spreading of naïve HCC1937 cells. Together, these results implicate a role of protein disulphide isomerases in supporting pro-migratory phenotypes of breast cancer cells and indicate a functional importance of PDIA3-dependent secreted products. Thus, PDIA3 activity may be significant for conditioning of the TME.

In comparing the activities of two inhibitors, we found that the phenotypic responses of MDA-MB-231 cells to 16F16 were markedly different from those to PACMA-31. Since PACMA-31 has a stronger inhibitory effect on PDIA1 than PDIA3 [33], whereas 16F16 inhibits both PDIA1 and PDIA3 [38], the distinctive effects of 16F16 may implicate PDIA3. However, the effects could be due to the additive effect of inhibiting PDIA1 and PDIA3, and 16F16 may potentially interact with other thioredoxin superfamily proteins including PDIA6 [39]. The use of CM from WT or *Pdia3^−/−^* MEF specifically indicated a requirement for PDIA3 for fibroblast-secreted products that are supportive of MDA-MB-231 breast cancer cell spreading and migration. In future, silencing by PDIA3 siRNA or CRISPR-mediated *PDIA3* gene-editing in these cell lines will be needed to clearly establish a role for PDIA3. The inhibitors used in these experiments have been shown to bind preferentially to PDIA1 or PDIA3, respectively, and are considered to be cell-permeable, colocalising in cells with intracellular PDIA1 (PACMA) or PDIA3 (16F16, [38]), each of which were reported to be located principally within the ER. Indeed, PDIA1 and PDIA3 protein sequences contain conserved C-terminal ER retention motifs. However, there are specific conditions and cell types for which extracellular PDIs have been reported, principally for PDIA1 [40,41] but also for other PDI including PDIA3 [35,42]. The precise location of PDIA3 in breast cancer cells is of interest for future research.

The reduction in F-actin structures observed in all three cell lines in response to 16F16 (relative to control conditions) implicates a role of protein disulphide isomerases in promoting pro-migratory phenotypes in these cells. A motile cancer cell attaches and spreads on glass in a similar manner to fibroblasts, forming pro-migratory F-actin structures including lamellipodia, ‘finger-like’ protrusions (including filopodia) and stress fibres [43]. Although the exact features of F-actin organisation were cell-line specific, overall, all these structures were present in control or PACMA31-treated cells and were reduced in 16F16-treated cells. These effects were most pronounced at later time points. This suggests a continuous role of protein disulphide isomerases in cell adhesion. Known PDIA3 substrates include integrin subunits, as well as certain ECM proteins including laminin subunits and FN [13,35], so the effects might be due to a reduction in cell-surface integrins (reducing cell attachment capacity) and/or reduced secretion of pro-adhesive ECM proteins (leading to reduced cell spreading). Since rounded cells attach more weakly than spread cells it is possible that a reduced number of attached cells could also be due to loss of rounded cells during the washing steps. The PACMA31 and 16F16 inhibitors have also been reported to inhibit attachment of MDA-MB-231 cells to endothelial monolayers and their trans-endothelial migration, which implicates roles of protein disulphide isomerases in cell–cell adhesion processes [44].

The alterations in cell areas and F-actin structures in 16F16-treated cells led to a prediction that 16F16 treatment would cause reduced cell motility. Indeed, for all three cell lines, 16F16-treatment reduced the cells’ ability to seal a ‘scratch wound’. For the basal-phenotype cell lines (MDA-MB-231 and HCC1937), 16F16 clearly had a greater inhibitory effect than PACMA-31. Because these ‘scratch-wound’ assays assess migration over a 2D surface, the setup may not be fully representative of *in vivo* migration; nevertheless, the mesenchymal migration of basal breast cancer cells might be favoured by a 2D environment [45–47]. Thus, the greater differences between conditions for the basal-like cell lines could be due to a greater baseline motility of these cells. However, contradicting this, the HCC1937 cells were the slowest to seal the wound. Because this experimental setup measures only ‘scratch closure’ and does not distinguish between migration and proliferation, it cannot be excluded that slower wound closure is in part due to decreased cell proliferation.

In view of the secreted or cell-surface ECM-associated proteins that have been shown to be reliant on the PDIA3–Calnexin–Calreticulin complex for secretion [13], the experiments with inhibitors were complemented by investigations of secreted products, analysed in the form of CM or ECM. CM from WT-MEF supported spreading and F-actin organisation in all three breast cancer cell lines, whereas CM from *Pdia3^−/−^* MEF conferred a phenotype similar to FGM in basal lines and reduced F-actin structures in MCF-7 cells. It should be noted that MCF-7 cells grew less well in FGM than in DMEM, probably due to the low level of Phenol Red in FGM: Phenol Red acts as an oestrogen mimetic that stimulates MCF-7 growth [48]. Thus, MCF-7 cells may be more sensitive to (for example) waste products in the CM, explaining why WT-CM did not accelerate cell spreading by these cells. Given that KO-CM clearly had reduced activity to support spreading and focal adhesion assembly in MDA-MB-231 cells, it was predictable that the KO-CM could not support ‘wound closure’ as effectively as FGM or WT-CM, although the initial rate of closure was not affected. These data imply that PDIA3-dependent secreted products of fibroblasts could have paracrine roles in enhancing migratory F-actin structures of breast cancer cells. This would be similar to the activities of cancer-associated fibroblasts [10,49]. Overall, the basal-like cell lines showed the strongest effects of pharmacological inhibition and the phenotypes of cells exposed to *Pdia3^−/−^* KO-CM were very similar to those resulting from 16F16 treatment. Further studies will be needed to identify the secreted proteins that differ between WT and *Pdia3^−/−^* MEF, which might include ECM proteins or chemokines that have known roles in tumour progression [50].

The role of secreted products that depend on protein disulphide isomerases’ activity for their folding and export was also examined using ECM from HCC1937 that had been produced under control or inhibited conditions. HCC1937 cells were chosen because they showed a clear response in the earlier experiments and are derived from a primary tumour and so represent pre-metastatic cells [28]. This was of particular interest as modifications to the ECM at the primary site are widely regarded as essential for metastasis initiation [51,52]. ECM from HCC1937 was highly supportive of attachment and spreading of HCC1937 cells. Within 1 h, cell areas were equivalent to the areas of cells plated for 24 h on glass. ECM produced under 16F16-treated conditions was significantly less supportive of cell attachment and spreading compared with fresh FGM. Whether this is due to specific alterations in ECM composition, or to reduced total ECM production in the presence of the inhibitor, remains for future determination. These data provide further support to the hypothesis that PDIA3 may have important roles in the production of a microenvironment supportive for breast cancer cell motility and invasion.

Overall, the data provide evidence for a role of protein disulphide isomerases including PDIA3 in promoting pro-migratory phenotypes in three breast cancer cell lines. The effects were most clear in basal-phenotype cells. We show that the functional activities can, at least in part, be attributed to PDIA3-dependent secreted products, from paracrine (fibroblast CM) sources, and possibly from autocrine (HCC ECM experiment) sources. These data provide new insight into functional roles of protein disulphide isomerases including PDIA3 that can impact breast cancer TME, which is known as a major driver of breast cancer metastasis. Given the current interest in blockade of extracellular proteins involved in host–tumour communications as possible new therapies for triple-negative breast cancer [53,54], it will be of future interest to extend this study to secreted products of human cancer-associated fibroblasts and to identify the secreted proteins that are altered under conditions of PDIA3 ablation.

## Supplementary Material

Supplementary Figures S1-S2

## Abbreviations

CM: conditioned media
DCIS: ductal carcinoma *in situ*
DMSO: dimethyl sulphoxide
ECM: extracellular matrix
EGF: epidermal growth factor
EMT: epithelial–mesenchymal transition
ER: endoplasmic reticulum
FGM: fibroblast growth medium
FN: fibronectin
GAPDH: glyceraldehyde 3-phosphate dehydrogenase
HRP: horseradish peroxidase
IDC: invasive ductal carcinoma
MEF: mouse embryonic fibroblast
PBS: phosphate buffered saline
PDIA1: protein disulphide isomerase A1
PDIA3: protein disulphide isomerase A3
PFA: paraformaldehyde
PVDF: polyvinylidene fluoride
ROI: region-of-interest
RT: room temperature
RWD: relative wound density
SDS/PAGE: sodium dodecyl sulphate/polyacrylamide gel electrophoresis
TME: tumour microenvironment
WT: wildtype

## References

[1] Cancer Research UK Breast cancer statistics UK, accessed Oct. 2019, http://www.cancerresearchuk.org/health-professional/cancer-statistics/statistics-by-cancer-type/breast-cancer

[2] Ferlay J., Soerjomataram I., Dikshit R., Eser S., Mathers C., Rebelo M. et al. (2015) Cancer incidence and mortality worldwide: sources, methods and major patterns in GLOBOCAN 2012. Int. J. Cancer 136, E359–E386 10.1002/ijc.29210 25220842

[3] Chaffer C.L. and Weinberg R.A. (2011) A perspective on cancer cell metastasis. Science 331, 1559–1564 10.1126/science.1203543 21436443

[4] Lambert A.W., Pattabiraman D.R. and Weinberg R.A. (2017) Emerging biological principles of metastasis. Cell 168, 670–691 10.1016/j.cell.2016.11.037 28187288 PMC5308465

[5] Redig A.J. and McAllister S.S. (2013) Breast cancer as a systemic disease: a view of metastasis. J. Intern. Med. 274, 113–126 10.1111/joim.12084 23844915 PMC3711134

[6] Wu Q., Yang Z., Nie Y., Shi Y. and Fan D. (2014) Multi-drug resistance in cancer chemotherapeutics: mechanisms and lab approaches. Cancer Lett. 347, 159–166 10.1016/j.canlet.2014.03.013 24657660

[7] Bong A.H.L. and Monteith G.R. (2017) Breast cancer cells: focus on the consequences of epithelial-to-mesenchymal transition. Int. J. Biochem. Cell Biol. 87, 23–26 10.1016/j.biocel.2017.03.014 28336365

[8] Chaffer C.L., San Juan B.P., Lim E. and Weinberg R.A. (2016) EMT, cell plasticity and metastasis. Cancer Metastasis Rev. 35, 645–654 10.1007/s10555-016-9648-7 27878502

[9] Broders-Bondon F., Nguyen Ho-Bouldoires T.H., Fernandez-Sanchez M.-E. and Farge E. (2018) Mechanotransduction in tumor progression: the dark side of the force. J. Cell Biol. 217, 1571–1587 10.1083/jcb.201701039 29467174 PMC5940296

[10] Gascard P. and Tlsty T.D. (2016) Carcinoma-associated fibroblasts: orchestrating the composition of malignancy. Genes Dev. 30, 1002–1019 10.1101/gad.279737.116 27151975 PMC4863733

[11] Roma-Rodrigues C., Mendes R., Baptista P.V. and Fernandes A.R. (2019) Targeting tumor microenvironment for cancer therapy. Int. J. Mol. Sci. 20, 840 10.3390/ijms20040840 PMC6413095 30781344

[12] Galligan J.J. and Petersen D.R. (2012) The human protein disulfide isomerase gene family. Hum. Genomics 6, 6–6 10.1186/1479-7364-6-6 23245351 PMC3500226

[13] Jessop C.E., Chakravarthi S., Garbi N., Hämmerling G.J., Lovell S. and Bulleid N.J. (2007) ERp57 is essential for efficient folding of glycoproteins sharing common structural domains. EMBO J. 26, 28–40 10.1038/sj.emboj.7601505 17170699 PMC1782378

[14] Frickel E.-M., Frei P., Bouvier M., Stafford W.F., Helenius A., Glockshuber R. et al. (2004) ERp57 is a multifunctional thiol-disulfide oxidoreductase. J. Biol. Chem. 279, 18277–18287 10.1074/jbc.M314089200 14871896

[15] Leys C.M., Nomura S., LaFleur B.J., Ferrone S., Kaminishi M., Montgomery E. et al. (2007) Expression and prognostic significance of prothymosin-alpha and ERp57 in human gastric cancer. Surgery 141, 41–50 10.1016/j.surg.2006.05.009 17188166

[16] Chung H., Cho H., Perry C., Song J., Ylaya K., Lee H. et al. (2013) Downregulation of ERp57 expression is associated with poor prognosis in early-stage cervical cancer. Biomarkers 18, 573–579 10.3109/1354750X.2013.827742 23957851

[17] Choe M.H., Min J.W., Jeon H.B., Cho D.-H., Oh J.S., Lee H.G. et al. (2015) ERp57 modulates STAT3 activity in radioresistant laryngeal cancer cells and serves as a prognostic marker for laryngeal cancer. Oncotarget 6, 2654–2666 10.18632/oncotarget.3042 25605256 PMC4413608

[18] Basu A., Cajigas-Du Ross C.K., Rios-Colon L., Mediavilla-Varela M., Daniels-Wells T.R., Leoh L.S. et al. (2016) LEDGF/p75 overexpression attenuates oxidative stress-induced necrosis and upregulates the oxidoreductase ERP57/PDIA3/GRP58 in prostate cancer. PLoS ONE 11, e0146549 10.1371/journal.pone.0146549 26771192 PMC4714844

[19] Ramos F.S., Serino L.T., Carvalho C.M., Lima R.S., Urban C.A., Cavalli I.J. et al. (2015) PDIA3 and PDIA6 gene expression as an aggressiveness marker in primary ductal breast cancer. Genet. Mol. Res. 14, 6960–6967 10.4238/2015.June.26.4 26125904

[20] Song M.N., Moon P.G., Lee J.E., Na M., Kang W., Chae Y.S. et al. (2012) Proteomic analysis of breast cancer tissues to identify biomarker candidates by gel-assisted digestion and label-free quantification methods using LC-MS/MS. Arch. Pharm. Res. 35, 1839–1847 10.1007/s12272-012-1018-6 23139137

[21] Da Costa G.G., Gomig T.H., Kaviski R., Santos Sousa K., Kukolj C., De Lima R.S. et al. (2015) Comparative proteomics of tumor and paired normal breast tissue highlights potential biomarkers in breast cancer. Cancer Genomics Proteomics 12, 251–261 26417028

[22] Wang J., Betancourt A.M., Mobley J.A. and Lamartiniere C.A. (2011) Proteomic discovery of genistein action in the rat mammary gland. J. Proteome Res. 10, 1621–1631 10.1021/pr100974w 21254785 PMC3070037

[23] Hussmann M., Janke K., Kranz P., Neumann F., Mersch E., Baumann M. et al. (2015) Depletion of the thiol oxidoreductase ERp57 in tumor cells inhibits proliferation and increases sensitivity to ionizing radiation and chemotherapeutics. Oncotarget 6, 39247–39261 10.18632/oncotarget.5746 26513173 PMC4770770

[24] Santana-Codina N., Carretero R., Sanz-Pamplona R., Cabrera T., Guney E., Oliva B. et al. (2013) A transcriptome-proteome integrated network identifies endoplasmic reticulum thiol oxidoreductase (ERp57) as a hub that mediates bone metastasis. Mol. Cell. Proteomics 12, 2111–2125 10.1074/mcp.M112.022772 23625662 PMC3734573

[25] Gaucci E., Altieri F., Turano C. and Chichiarelli S. (2013) The protein ERp57 contributes to EGF receptor signaling and internalization in MDA-MB-468 breast cancer cells. J. Cell. Biochem. 114, 2461–2470 10.1002/jcb.24590 23696074

[26] Wise R., Duhachek-Muggy S., Qi Y., Zolkiewski M. and Zolkiewska A. (2016) Protein disulfide isomerases in the endoplasmic reticulum promote anchorage-independent growth of breast cancer cells. Breast Cancer Res. Treat. 157, 241–252 10.1007/s10549-016-3820-1 27161215 PMC5662471

[27] Tomlinson G.E., Chen T.T.L., Stastny V.A., Virmani A.K., Spillman M.A., Tonk V. et al. (1998) Characterization of a breast cancer cell line derived from a germ-line *BRCA1* mutation carrier. Cancer Res. 58, 3237 9699648

[28] Cailleau R., Young R., Olivé M. and Reeves J.W.J. (1974) Breast tumor cell lines from pleural effusions. J. Natl. Cancer Inst. 53, 661–674 10.1093/jnci/53.3.661 4412247 PMC7364228

[29] Soule H.D., Vazquez J., Long A., Albert S. and Brennan M. (1973) A human cell line from a pleural effusion derived from a breast carcinoma. J. Natl. Cancer Inst. 51, 1409–1416 10.1093/jnci/51.5.1409 4357757

[30] Coe H., Jung J., Groenendyk J., Prins D. and Michalak M. (2010) ERp57 modulates STAT3 signaling from the lumen of the endoplasmic reticulum. J. Biol. Chem. 285, 6725–6738 10.1074/jbc.M109.054015 20022947 PMC2825467

[31] Hellewell A.L., Rosini S. and Adams J.C. (2017) A rapid, scalable method for the isolation, functional study, and analysis of cell-derived extracellular matrix. J. Vis. Exp. 119, 10.3791/55051 PMC5351878 28117783

[32] Xu S., Butkevich A.N., Yamada R., Zhou Y., Debnath B., Duncan R. et al. (2012) Discovery of an orally active small-molecule irreversible inhibitor of protein disulfide isomerase for ovarian cancer treatment. Proc. Natl. Acad. Sci. U.S.A. 109, 16348–16353 10.1073/pnas.1205226109 22988091 PMC3479552

[33] Bekendam R.H., Bendapudi P.K., Lin L., Nag P.P., Pu J., Kennedy D.R. et al. (2016) A substrate-driven allosteric switch that enhances PDI catalytic activity. Nat. Commun. 7, 12579 10.1038/ncomms12579 27573496 PMC5013553

[34] Hoffstrom B.G., Kaplan A., Letso R., Schmid R., Turmel G.J., Lo D.C. et al. (2010) Inhibitors of protein disulfide isomerase suppress apoptosis induced by misfolded proteins. Nat. Chem. Biol. 6, 900–906 10.1038/nchembio.467 21079601 PMC3018711

[35] Dihazi H., Bibi A., Eltoweissy M., Mueller C.A., Asif A.R., Rubel D. et al. (2013) Secretion of ERP57 is important for extracellular matrix accumulation and progression of renal fibrosis, and is an early sign of disease onset. J. Cell Sci. 126, 3649–3663 10.1242/jcs.125088 23781031

[36] Son H. and Moon A. (2010) Epithelial-mesenchymal transition and cell invasion. Toxicol. Res. 26, 245–252 10.5487/TR.2010.26.4.245 24278531 PMC3834497

[37] Trepat X., Chen Z. and Jacobson K. (2012) Cell migration. Compr. Physiol. 2, 2369–2392 23720251 10.1002/cphy.c110012 PMC4457291

[38] Ge J., Zhang C.J., Li L., Chong L.M., Wu X., Hao P. et al. (2013) Small molecule probe suitable for in situ profiling and inhibition of protein disulfide isomerase. ACS Chem. Biol. 28, 2577–2585 10.1021/cb4002602 24070012

[39] Foster C.K. and Thorpe C. (2017) Challenges in the evaluation of thiol-reactive inhibitors of human protein disulfide isomerase. Free Radic. Biol. Med. 108, 741–749 10.1016/j.freeradbiomed.2017.04.367 28465261 PMC5507595

[40] Bekendam R.H. and Flaumenhaft R. (2016) Inhibition of protein disulfide isomerase in thrombosis. Basic Clin. Pharmacol. Toxicol. 119, 42–48 10.1111/bcpt.12573 26919268

[41] Cho J., Furie B.C., Coughlin S.R. and Furie B. (2008) A critical role for extracellular protein disulfide isomerase during thrombus formation in mice. J. Clin. Invest. 118, 1123–1131 18292814 10.1172/JCI34134 PMC2248441

[42] Holbrook L.-M., Sasikumar P., Stanley R.G., Simmonds A.D., Bicknell A.B. and Gibbins J.M. (2012) The platelet-surface thiol isomerase enzyme ERp57 modulates platelet function. J. Thromb. Haemost. 10, 278–288 10.1111/j.1538-7836.2011.04593.x 22168334 PMC3444690

[43] Yamaguchi H. and Condeelis J. (2007) Regulation of the actin cytoskeleton in cancer cell migration and invasion. Biochim. Biophys. Acta 1773, 642–652 10.1016/j.bbamcr.2006.07.001 16926057 PMC4266238

[44] Popielarski M., Ponamarczuk H., Stasiak M., Watała C. and Świątkowska M. (2019) Modifications of disulfide bonds in breast cancer cell migration and invasiveness. Am. J. Cancer Res. 9, 1554–1582 31497343 PMC6727000

[45] Bertucci F., Finetti P. and Birnbaum D. (2012) Basal breast cancer: a complex and deadly molecular subtype. Curr. Mol. Med. 12, 96–110 10.2174/156652412798376134 22082486 PMC3343384

[46] Friedl P., Locker J., Sahai E. and Segall J.E. (2012) Classifying collective cancer cell invasion. Nat. Cell Biol. 14, 777–783 10.1038/ncb2548 22854810

[47] Friedl P. and Wolf K. (2010) Plasticity of cell migration: a multiscale tuning model. J. Cell Biol. 188, 11–19 10.1083/jcb.200909003 19951899 PMC2812848

[48] Welshons W.V., Wolf M.F., Murphy C.S. and Jordan V.C. (1988) Estrogenic activity of phenol red. Mol. Cell. Endocrinol. 57, 169–178 10.1016/0303-7207(88)90072-X 3402660

[49] Erdogan B., Ao M., White L.M., Means A.L., Brewer B.M., Yang L. et al. (2017) Cancer-associated fibroblasts promote directional cancer cell migration by aligning fibronectin. J. Cell Biol. 216, 3799–3816 10.1083/jcb.201704053 29021221 PMC5674895

[50] Bikfalvi A. and Billottetm C. (2020) The CC and CXC chemokines: major regulators of tumor progression and the tumor microenvironment. Am. J. Physiol. Cell Physiol. 318, C542–C554 10.1152/ajpcell.00378.2019 31913695 PMC7099520

[51] Kaushik S., Pickup M.W. and Weaver V.M. (2016) From transformation to metastasis: deconstructing the extracellular matrix in breast cancer. Cancer Metastasis Rev. 35, 655–667 10.1007/s10555-016-9650-0 27914000 PMC5215979

[52] Hu G., Li L. and Xu W. (2017) Extracellular matrix in mammary gland development and breast cancer progression. Front. Lab. Med. 1, 36–39 10.1016/j.flm.2017.02.008

[53] Buchsbaum R.J. and Oh S.Y. (2016) Breast cancer-associated fibroblasts: where we are and where we need to go. Cancers (Basel) 8, 19 10.3390/cancers8020019 PMC4773742 26828520

[54] Jin K., Pandey N.B. and Popel A.S. (2018) Simultaneous blockade of IL-6 and CCL5 signaling for synergistic inhibition of triple-negative breast cancer growth and metastasis. Breast Cancer Res. 20, 54–54 10.1186/s13058-018-0981-3 29898755 PMC6000947

