# Supplementary material for: Impairment of cell adhesion and migration by inhibition of protein disulphide isomerases in three breast cancer cell lines
Source: Biosci Rep. 2020 Oct 23;40(10):BSR20193271. doi: 10.1042/BSR20193271 (PMC7584814; doi:10.1042/BSR20193271)
Supplement: Supplementary Figures S1-S2 [file BSR-2019-3271_supp.pdf]

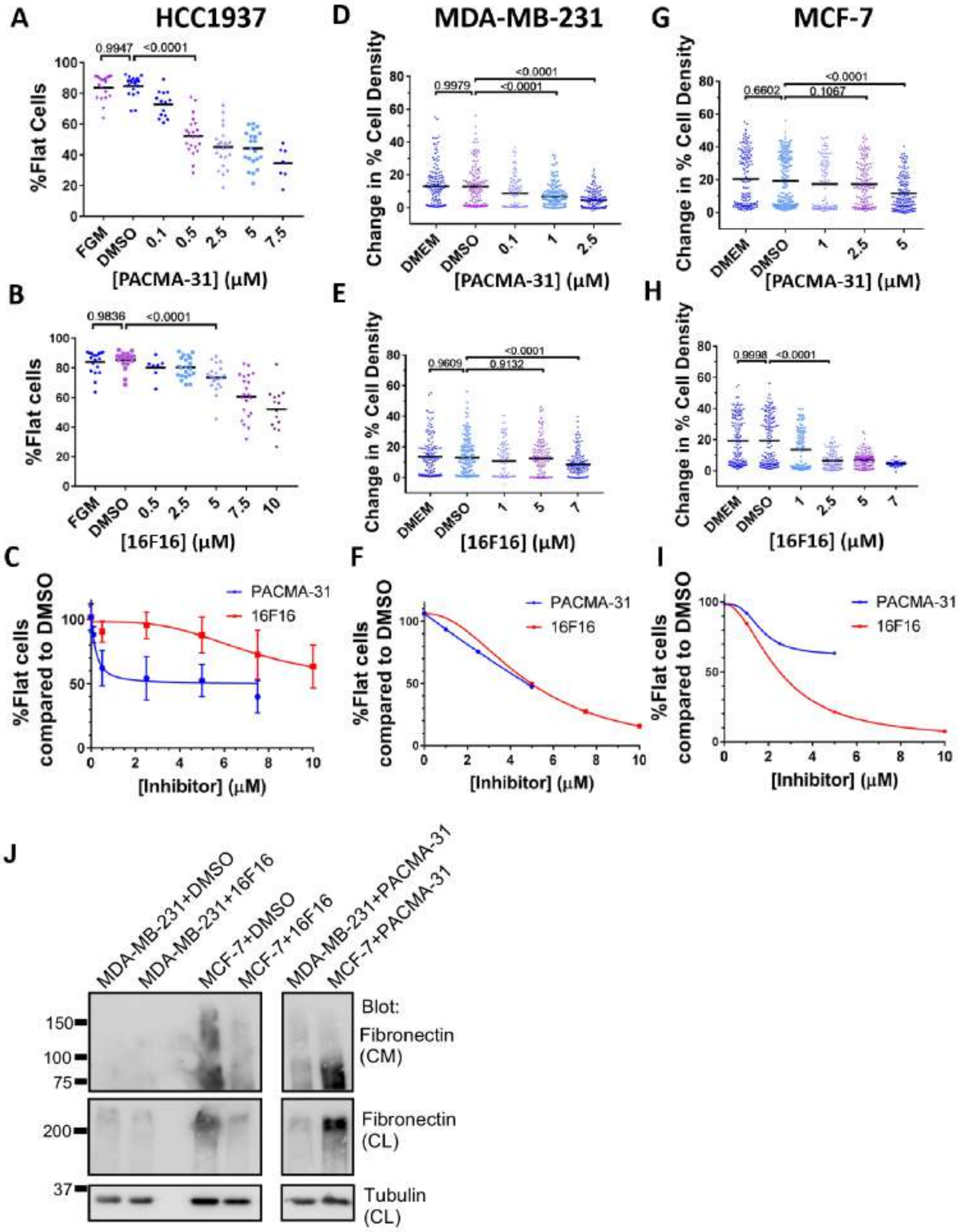

Supplementary Figure 1. Concentration-dependent effects of PACMA-31 and 16F16 inhibitors on spreading of human breast cancer cell lines. HCC1937 (A, B) were treated with the indicated concentrations of PACMA-31 (A) or 16F16 (B) for x h, washed, fixed and scored for % of cells retaining a flat morphology. D-H, effects on cell density of MDA-MB-231 cells (D, E) or MCF-7 cells (G,H), scored from IncuCyte cell confluence data, after treatment with PACMA-31 (D, G) or 16F16 (E, H). In A-H, each dot represents a single well and bars show the median. C-I, data from HCC1937 (C), MDA-MB-231 (F) or MCF-7 (I) cells presented as mean +/- SD relative to +DMSO values. J, Blot for fibronectin in cell layer (CL) or conditioned media (CM) of MDA-MB-231 or MCF-7 cells under the indicated conditions. a-Tubulin was used as a loading control. Markers are in kDa.

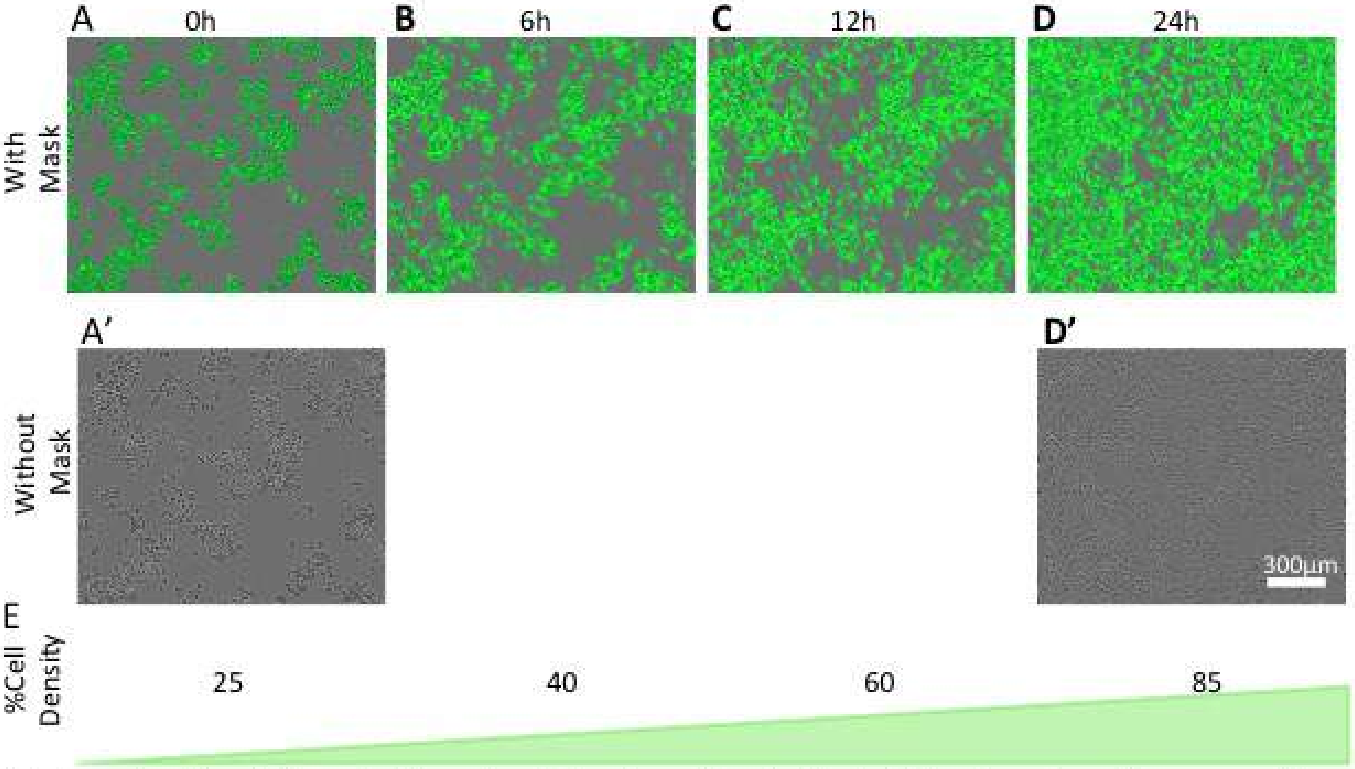

Supplementary Figure 2. Example of the automated masking achieved by the IncuCyte software from a time course with MDA-MB-231 cells. A-D, phase contrast images of cells at different time points as indicated, with the IncuCyte software masks applied. A′, the phase contrast image only from 0h time point that is shown in A. D′, the phase contrast image only from the 24h time point that is shown in D. E, schematic to show the automatically calculated % cell density from each masked image (in A-D).
